# Functions and Diseases of the Retinal Pigment Epithelium

**DOI:** 10.3389/fphar.2021.727870

**Published:** 2021-07-28

**Authors:** Song Yang, Jun Zhou, Dengwen Li

**Affiliations:** ^1^ State Key Laboratory of Medicinal Chemical Biology, College of Life Sciences, Nankai University, Tianjin, China; ^2^ Institute of Biomedical Sciences, Shandong Provincial Key Laboratory of Animal Resistance Biology, Collaborative Innovation Center of Cell Biology in Universities of Shandong, College of Life Sciences, Shandong Normal University, Jinan, China

**Keywords:** retina, retinal pigment epithelium, development, function, disease, retinopathy, therapy

## Abstract

The retinal pigment epithelium is a fundamental component of the retina that plays essential roles in visual functions. Damage to the structure and function of the retinal pigment epithelium leads to a variety of retinopathies, and there is currently no curative therapy for these disorders. Therefore, studying the relationship between the development, function, and pathobiology of the retinal pigment epithelium is important for the prevention and treatment of retinopathies. Here we review the function of the retinal pigment epithelium and its relevance to the pathobiology, and discuss potential strategies for the treatment of retinopathies. In doing so, we provide new viewpoints outlining new ideas for the future study and treatment of retinopathies.

## Introduction

Retinal pigment epithelium (RPE) is formed from a single layer of regular polygonal cells arranged at the outermost layer of the retina. The outer side of the RPE is connected to Bruch’s membrane and the choroid, while the inner side is connected to the outer segment of photoreceptor cells. The outer side exhibits basal infolding, which increases cell surface area and facilitates substance exchange. The basement membrane is closely connected to the basal folds by half desmosomes located in the innermost layer of Bruch’s membrane. The inside of RPE cells harbors microvillous structures extending between photoreceptor outer segments (POS), which participate in the phagocytic function of the RPE (Song and Zhou, 2020; Zhou and Zhou, 2020; Yang et al., 2021). The tight junction formed between the single-layer RPE and the gap junction control the movement of substances and at the same time forms the choroid-blood-retinal barrier with Bruch’s membrane and choroid at the lateral retina (Xie et al., 2020). The RPE appears dark brown due to its melanin content, which reduces damage to the retina and internal nerves from ultraviolet light (Tian et al., 2021). The RPE also harbors a complex metabolic system that reduces excessive accumulation of reactive oxygen species (ROS) and consequent oxidative damage.

Therefore, RPE structure and function are essential to normal vision, and alterations in the RPE can impair function and lead to retinopathy. For example, retinitis pigmentosa (RP), age-related macular degeneration (AMD), and Stargardt disease (SD) are degenerative retinal diseases in which RPE dysfunction has been implicated in their pathogenesis [for an excellent review, see Zarbin (Zarbin, 2016)]. RP afflicts 100,000 people in the United States and usually causes visual loss in childhood or young adulthood. AMD afflicts 1.75 million people in the United States alone, is the leading cause of blindness in individuals over 55 years of age in the United States and Europe, and was estimated to affect ∼196 million people worldwide in 2020. SD is the most common form of inherited juvenile macular degeneration, with a prevalence of 1 in 10,000 births. There are currently no cures for these degenerative diseases, so understanding the role of RPE in their pathogenesis is important for the development of new approaches to manage these common and debilitating disorders. Here we review the functions and diseases of the RPE to provide a theoretical basis for the treatment and prevention of associated diseases.

## Retinal Development and Structure

The human eye begins to develop at embryonic day (E)18. The visual groove is formed at E22 before continuing to sag to form the visual vesicle, which expands to form the inner and outer layers of the optic cup. The RPE layer begins to differentiate around E30, with pigment particles found in RPE cells at E35. A set of genes (including *PAX6*, *LHX2*, *RAX*, and *SIX3*) expressed in the neural plate before E8 are involved in eye determination and eventually form the optic cup (Hoon et al., 2014). In general, vertebrate RPE cells develop and differentiate from optic vesicles. During embryonic development, early optic vesicle cells have bidirectional potential and can develop into the retinal neurocortical layer or the RPE layer. The cell fate decision and differentiation of RPE precursor cells is not spontaneous but rather influenced by a variety of microenvironmental factors. Under the influence of extracellular signals, differentiation is guided in strict temporal and spatial order through the regulation of transcription factors and intracellular signaling pathways. In particular, the transcription factor MITF (microphthalmia-associated transcription factor) has been confirmed to be involved in the normal RPE development, and *Mitf* knockout results in abnormal retinal development in mice (Bharti et al., 2008; Ma et al., 2019).

**FIGURE 1:**
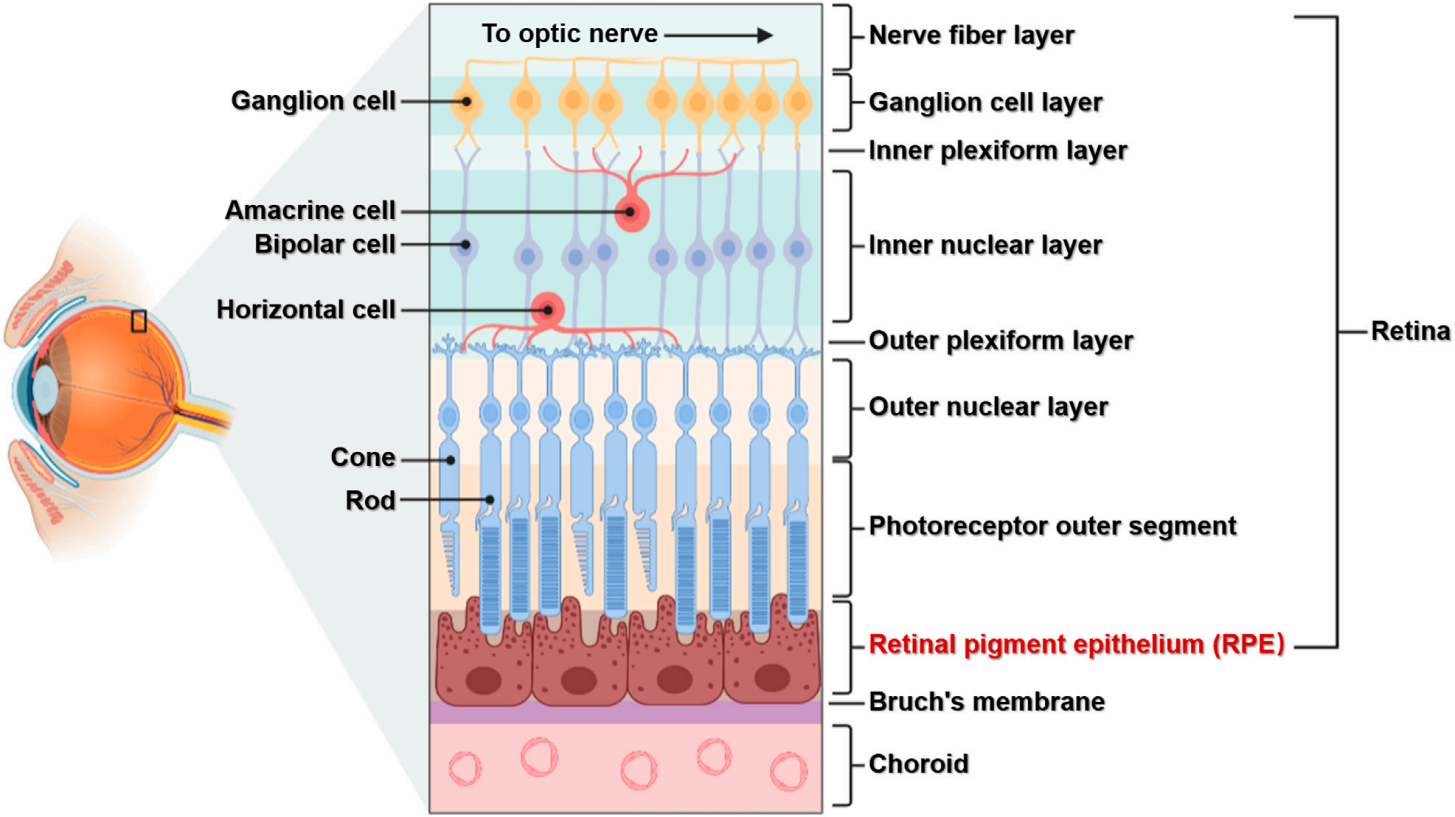
**The structure of the retina.** The retina is composed of multiple layers and different cell types. The RPE is composed of a single layer of RPE cells, which are connected to the choroid membrane through Bruch’s membrane.

Light entering the eye is focused on the retina, which converts light signals into electrical signals that travel through the optic nerve to the visual center of the brain (Grossniklaus et al., 2015). The retina is located in the fundus of the eye and, as an important tissue forming vision, has a complex structure. The retina has multiple layers containing various cell types: the RPE lies at the boundary, while the retinal nerve layer contains five main neuronal cells including photoreceptor cells (rods and cones), horizontal cells, bipolar cells, amacrine cells, and ganglion cells. RPE cells are located between photoreceptor cells and the choroid membrane, with the basal side connected to Bruch’s membrane and tip microvilli connected to the outer segment of photoreceptor cells (Figure 1). The RPE is located in a specific position and has important functions, and the cells have no regenerative potential (Masland, 2012; Silverman and Wong, 2018). Therefore, studying the relationships between its structure, function, and associated diseases is important for the prevention and treatment of RPE lesions.

## Function of the Retinal Pigment Epithelium

### Maintaining the Visual Cycle and Phagocytosis

**FIGURE 2:**
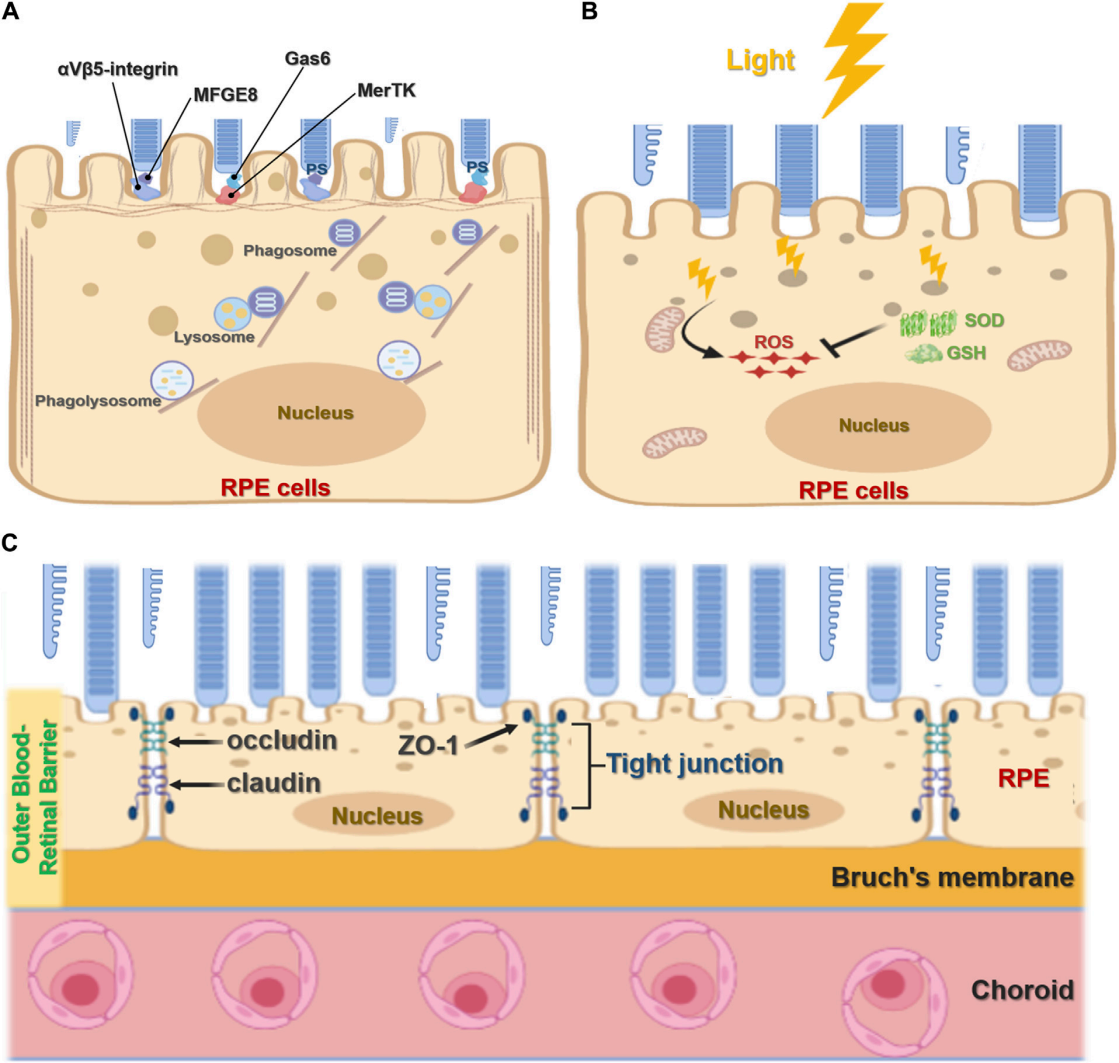
**Function of retinal pigment epithelial cells. (A)**, The phagocytic function of RPE cells. RPE cells recognize and bind phosphatidylserine (PS) exposed by POS through MerTK/Gas6 and αVβ5-integrin/MFGE8 pathways to initiate phagocytosis. It further forms phagosomes and binds with lysosomes to form the phagolysosome, which digests POS. **(B)**, Antioxidant function of RPE cells. Light stress produces ROS. RPE cells absorb light through melanin or eliminate ROS accumulation through antioxidants such as superoxide dismutase (SOD) and glutathione (GSH). **(C)**, RPE cell barrier function. The RPE forms an outer blood-retinal barrier between the interior of the retina and the choroid. The RPE cells form tight junctions, including ZO-1, occludin, and claudin, which act as barriers.

The RPE plays an important role in maintaining visual function and the visual cycle. RPE cells are phagocytic, with the ability to engulf and eliminate exfoliated POS and maintain the normal renewal of visual cells (Ran et al., 2020; Ran and Zhou, 2020). In mammals, each RPE cell is responsible for about 30 photoreceptors, and of all cell types RPE cells consume the most material in a mammal’s lifetime (Young, 1967; Young, 1971; Penberthy et al., 2018). RPE cell phagocytosis is divided into three stages: binding, endocytosis, and elimination. During binding, the inner microvilli cell membranes of RPE cells bind to the shed outer segment of the visual cell before being endocytosed into the cell and finally being transported by the cytoskeleton and vesicles to lysosomes for elimination. The TAM receptor tyrosine kinase MerTK is expressed by RPE cells and is crucial for RPE function, mediating the recognition and endocytosis of the POS by RPE cells. RPE cells without MerTK cannot engulf the POS, causing complete degeneration of the photoreceptor and blindness after birth (Prasad et al., 2006; Burstyn-Cohen et al., 2012). Other studies have shown that mice lacking αvβ5 integrin have gradually reduced retinal phagocytic capacity with age (Nandrot et al., 2004; C. Yu et al., 2019a) *via* a mechanism by which αvβ5 integrin acts as a receiver for the POS (Figure 2A). In addition, the *RPE65* gene encodes all-trans retinol ester isomerase, which is essential for the retinoid cycle. Mutation of the *RPE65* allele has been found to destroy optic cells and cause clinical manifestations of Leber congenital amaurosis type 2 (LCA2) and early-onset retinal dystrophy, eventually leading to complete blindness (Gu et al., 1997; Marlhens et al., 1997; Aguirre et al., 1998).

### Protection and Anti-Oxidative Functions

Located in the outermost layer of the retina, the RPE is rich in pigment particles including melanin and lipofuscin, which prevent light damage. These pigment particles are formed *in utero* and are no longer synthesized after birth. RPE melanin absorbs and filters natural light and protects the neural parts of the retina. Since the eyes are exposed to various light stimuli, they exist in a physiological state of photooxidation, accumulating high levels of oxygen free radicals that threaten oxidative damage. As a result, RPE cells contain many antioxidants such as superoxide dismutase and glutathione (Figure 2B). Melanosomes also participate in the antioxidant process, scavenging oxygen free radicals. Several mechanisms have been shown to underpin the antioxidant capacity and regulation of RPE cells including the ERK signaling pathway (Chong and Zheng, 2016; Chen et al., 2021); MMP-14 and TIMP-2 (Alcazar et al., 2007); micro(mi)RNA-23 (Lin et al., 2011); and toll-like receptor 3 (TLR3) (Patel and Hackam, 2013).

### Barrier and Substance Transport Functions

RPE cells are terminally differentiated in a mitotic quiescent phase. As a typical barrier cells, they guard both the inside and outside of the retina and strictly control substance entry and exit. RPE cells form tight junctions through ZO-1, occludin, and claudin, acting as the outer blood barrier between choroidal pore capillaries and the retinal photosensitive layer (Figure 2C). RPE cells use membrane pumps, endocytosis, passive diffusion, and other mechanisms to complete transport and play a key role in nutrient, water, and electrolyte transport between the choroid and retinal cells (Danesh-Meyer et al., 2016; Sun and Zhou, 2020). To achieve epithelium-specific functions, tight junction permeability and selectivity must match the epithelium-specific extracellular transport mechanism. At the same time, the rich membrane pump system in the retina, including the Na-K-ATPase system, enables ion concentrations on both sides to reach a dynamic balance and maintain normal retinal function. In addition, the rich RPE cell transporter system facilitates the transport of substances inside and outside the retina (Sugasawa et al., 1994).

## Retinal Pigment Epithelial Diseases and Pathogenesis

### Oxidative Stress and Inflammation

The cornea has a transparent structure and the RPE is exposed to light for long periods of time, has a rich oxygen supply, and consequently large amounts of reactive oxygen are easily generated. In addition, increased systemic glucose levels, such as in diabetics, can facilitate excessive ROS accumulation (F. Yu et al., 2019b; Yu et al., 2020). In degenerative retinopathy, antioxidant levels decrease in cells; that is, the capacity of RPE cells to remove ROS variably reduces, resulting in a large accumulation of POS (Mitter et al., 2014; Campello et al., 2020). Many studies have been conducted on retinal epithelial damage caused by oxidative stress and inflammation, and recent studies have shown that the redox-sensitive microRNA (miR)-144 plays an important role in the regulation of antioxidant signaling pathways in human and mouse RPE. Oxidative stress enhanced the expression of miR-144-3p and mir-144-5p, decreased expression of *Nrf2* and downstream antioxidant target genes *Nqo1* and *Gclc*, decreased glutathione levels, and increased RPE cell death (Yam et al., 2019; Jadeja and Martin, 2020). In summary, oxidative stress and inflammation cause RPE cell damage, which in turn causes retinal dysfunction and even blindness.

### Apoptosis and Autophagy

AMD is a serious neurodegenerative disease and a major cause of blindness in developed countries. Transcriptional profiling has shown that diseased RPE exhibits increased apoptosis, autophagy, and endoplasmic reticulum stress levels than normal cells. Other studies have shown that retinopathy is associated with disrupted cellular homeostasis and increased apoptosis, endoplasmic reticulum stress, and autophagy. Moreover, RPE cell death *via* apoptosis and endoplasmic reticulum stress has been observed in AMD and other retinal degenerative diseases (Dunaief et al., 2002; Feher et al., 2006; Bazan, 2007). In a study of RPE cells cultured *ex vivo* from AMD and normal donors, RPE cells from AMD donors accumulated lipid droplets and glycogen particles, harbored disintegrated mitochondria, and had increased numbers of autophagosomes. In addition, compared to RPE cells cultured from normal donors, RPE cells from AMD donors showed increased sensitivity to oxidative stress and decreased mitochondrial activity. The impaired autophagy function of AMD donor RPE was also demonstrated through measurement of the ratio of autophagy markers LC3-II/LC3-I (Golestaneh et al., 2017). These findings indicate a potential pathological mechanism for AMD through abnormal apoptosis and autophagy, thereby providing new targets for novel therapeutic strategies.

### Cell Polarity and Interactions

The polarity and cell junctions of the RPE play critical roles in the blood-retinal barrier, maintaining the stability of the internal photoreceptor microenvironment and supporting the choroidal system. Disrupted cell polarity and cell junctions significantly increase the risk of retinal degenerative disease (Caceres and Rodriguez-Boulan, 2020). RPE polarity and cell junction stability are related to the unique basal and apical structures of the retina, which affect phagocytosis and material exchange. For example, cholesterol efflux is mediated by the ABCA1 transport protein at the top and basolateral aspects of the cell (Storti et al., 2017). RPE phagocytic defects are related to photoreceptor degeneration, so further study of the RPE endocytic pathway may help establish new mechanisms of retinal diseases (Anderson et al., 2017; Kaur et al., 2018; Ran et al., 2021). In addition, patients with RP have been shown to have RPE polarity and functional defects, and the cilial mRNA splicing factor *PRPF* was mutated in these RPE cells (Buskin et al., 2018). In summary, abnormalities in RPE polarity, barrier destruction, and retinal stability may contribute to the pathogenesis of blinding retinal diseases.

## Treatment of Retinal Pigment Epithelial Diseases

### Cell Therapy

Stem cell-derived RPE and photoreceptors have restored vision in pre-clinical models of human retinal degenerative diseases. Stem cell transplantation may therefore be an effective future approach to treat RPE diseases. The sources of cells used for retinal cell therapy include stem cells such as embryonic stem cells (ESCs), adult stem cells, and induced pluripotent stem cells (iPSCs). Currently, ESCs, and iPSCs are mainly used for differentiation into RPE, but these cell types still have some limitations including allogeneic rejection and carrying donor pathogenic genes. Stem cell transplantation is feasible for the treatment of retinal epithelial lesions (Zamiri et al., 2006). Indeed, allogeneic fetal retina-RPE transplants under the retina were not rejected in people with RP and advanced AMD (Enzmann et al., 1999). However, the immune privilege of the RPE is not absolute (Zamiri et al., 2004). In another study, patients with AMD undergoing CNV resection received subretinal allogeneic RPE transplantation, and there was immune rejection after immunosuppressive therapy was stopped (Tezel et al., 2007). The clinical application of cell therapy for retinal degenerative diseases faces some important challenges including cell manufacturing, delivery, survival, and physiological behavior; immune responses; and a risk of cancer development.

### Gene and Drug Therapy

Thanks to many years of research into retinal diseases, many genes and signal transduction pathways have been identified as potential targets for gene therapy or other therapeutics. For example, ITH12674 is a melatonin and sulforaphane hybrid drug that induces expression of the transcription factor *Nrf2*, which can alleviate retinal degeneration leading to blindness (Campello et al., 2020). The lipid molecule ELV blocks the CB1 receptor and PLD2 in the eye to delay the development of degenerative and inflammatory retinal pathology (Bermudez et al., 2019). Emixustat is a non-retinal small molecule hydrochloride that acts as a highly efficient and selective visual cycle modulator targeting visual cycle isomerase. In the AMD animal model, emixustat can reduce A2E levels, protect the retina from light-mediated damage, and reduce neovascularization in premature retinopathy models (Kubota et al., 2020). Humanin (HNG), a 2.7 kDa 24 amino acid polypeptide, was discovered in a cDNA library derived from the brains of patients with familial Alzheimer’s disease. HNG protects primary RPE cells from oxidative damage (Nashine et al., 2017). Retinal cell mitochondria are severely damaged in AMD patients, and HNG is an important cell survival factor that can protect ARPE-19 RPE cell line mitochondria, making it an exciting target in AMD (Gong et al., 2018). In addition, as the first approved target for ophthalmological treatment, recombinant adeno-associated viruses (AAVs) have been used to deliver the *RPE65* gene into RPE cells with mutations or absence of RPE65 to prevent and treat inherited retinal diseases, such as LCA2 and inherited retinal dystrophy (Acland et al., 2001; Russell et al., 2017).

## Outlook

The RPE promotes normal retinal function and is an integral part of the retinal system, arising at the earliest stage of retinal development. As specialized phagocytes, RPE cells undertake the most intensive phagocytic task in the body through POS phagocytosis and maintaining the normal renewal and function of rod and cone cells. Its function in the blood-retinal barrier plays an important role in nutrient transport, maintenance of ion homeostasis, and the steady state of the microenvironment inside and outside the retina. The excessive accumulation of ROS and consequent retinopathy due to RPE dysfunction mean that these cells remain an important research focus. The visual circulation maintenance, barrier, substance transport, protection, and antioxidant functions of the RPE are all impaired to varying degrees in retinal degenerative diseases, highlighting the importance and necessity to protect the RPE and maintain or restore its function. Multiple mutations associated with AMD have been identified using sequencing techniques of different strategies. The genes in the complement system, such as complement C3 (C3), complement C9 (C9), complement factor I (CFI), and complement factor H (CFH), are associated with the development of AMD. When the membrane cofactor protein CD46 was knocked out, the mice developed lesions consistent with human dry AMD. In addition, mutations in the promoter of the *HTRA1* (high-temperature requirement protein A1) gene lead to increased HTRA1 expression, resulting in the occurrence of AMD. *FGD6* (FYVE, Rho GEF and PH domain-containing 6) gene mutation leads to increased expression of HTRA1 protein, which increases the risk of wet AMD (Lyzogubov et al., 2016; de Breuk et al., 2020).

At present, there is no effective and feasible method for the treatment of retinal degenerative diseases, although our increased understanding of the cellular biology and molecular genetics of retinal diseases might provide new avenues for prevention and treatment. Given the importance of the RPE in normal retinal function, this cell type provides an excellent focus for the development of treatments for retinopathy. In that regard, stem cell transplantation and gene therapy are currently hot research topics. While directing stem cell differentiation into RPE cells to restore function is an exciting and promising research direction, application to humans might be hampered by poor efficacy or immune reactions; further research is necessary. Encouragingly, as the study of the genes involved in the development of retinopathy and their signaling pathways increases, various inhibitors designed to target pathogenic genes and mutations are emerging, particularly for the treatment of RP. However, it still remains to be determined whether these targets are applicable to humans and whether modulation of these signaling pathways has off-target effects that might result in adverse reactions.
